# Views on electronic cigarette use in tobacco screening and cessation in an Alaska Native healthcare setting

**DOI:** 10.3402/ijch.v74.27794

**Published:** 2015-10-19

**Authors:** Vanessa Y. Hiratsuka, Jaedon P. Avey, Susan B. Trinidad, Julie A. Beans, Renee F. Robinson

**Affiliations:** 1 Research Department, Southcentral Foundation, Anchorage, AK, USA; 2 Department of Bioethics and Humanities, University of Washington School of Medicine, Seattle, WA, USA

**Keywords:** electronic cigarettes, qualitative research, tobacco use, Indians, North America

## Abstract

**Background:**

American Indian (AI) and Alaska Native (AN) communities confront some of the highest rates of tobacco use and its sequelae.

**Methods:**

This formative research project sought to identify the perspectives of 41 stakeholders (community members receiving care within the healthcare system, primary care providers, and tribal healthcare system leaders) surrounding the use of pharmacogenetics toward tobacco cessation treatment in the setting of an AI/AN owned and operated health system in south central Alaska.

**Results:**

Interviews were held with 20 adult AI/AN current and former tobacco users, 12 healthcare providers, and 9 tribal leaders. An emergent theme from data analysis was that current tobacco screening and cessation efforts lack information on electronic cigarette (e-cigarette) use. Perceptions of the use of e-cigarettes role in tobacco cessation varied.

**Conclusion:**

Preventive screening for tobacco use and clinical cessation counseling should address e-cigarette use. Healthcare provider tobacco cessation messaging should similarly address e-cigarettes.

Globally, the electronic cigarette (e-cigarette) or electronic nicotine delivery system (ENDS) has grown as a product with unknown health effects (1). ENDS represent a new frontier in tobacco control as the products offer individuals an opportunity to obtain nicotine while not inhaling or exhaling tobacco smoke (2). Across the circumpolar north, e-cigarettes and e-liquids which are used in ENDS have variable restrictions governing distribution. For instance, nicotine e-cigarettes are not authorized for sale in Canada (3) yet in Finland and the United States national laws prohibit the sale of tobacco products to minors but do not regulate nicotine e-cigarette sales (1, 4). Knowledge, attitudes, beliefs and behaviours around ENDS use among circumpolar indigenous populations are not well described despite high indigenous tobacco use rates.

Tobacco use is the leading cause of preventable illness and death in the United States (US) with American Indian (AI) and Alaska Native (AN) people having higher tobacco use rates than any other US racial/ethnic group (5–11). Although the average number of cigarettes smoked per day is lower among AN smokers than among US White smokers, tobacco-related disease burden is higher (12–14). Neither AI/AN views on e-cigarette/ENDS use among the AI/AN population, nor AI/AN views on e-cigarettes/ENDS are well described.

An e-cigarette is a battery-powered device that aerosolizes a solution, often containing nicotine, by heat (15). Individuals who regularly use e-cigarettes often refer to themselves as “vapers” as opposed to smokers, and talk about e-cigarette use as “vaping” rather than smoking. E-cigarettes were introduced in the US in 2007, and their popularity has grown considerably (16). Television advertising and e-cigarette cartridge flavours such as bubble gum appear to target the younger population.

E-cigarette use is assumed to be less dangerous than traditional tobacco cigarettes, but supporting data are lacking (16–20). Depending on the ENDS apparatus used, carcinogenic content of the aerosolized e-liquid can be comparable to that of tobacco smoke (16). The World Health Organization classifies e-cigarettes in the same category as smokeless tobacco and considers e-cigarette use a public health risk (18, 21). Currently, a small number of state and local governments have enacted policies to regulate e-cigarette sales and use, but federal regulations in the US do not exist. E-cigarettes are advertised and typically viewed as a competitor to existing smoking cessation methods (e.g. nicotine gum, nicotine patch) (16, 18, 20, 21). A limited number of studies have displayed e-cigarettes’ capability to aid in reducing cigarette use, but no robust data exist that support the use of e-cigarettes as a smoking cessation aid (15).

Few studies have examined factors influencing tobacco use and tobacco cessation in the AI/AN population. Understanding the knowledge, attitudes, and beliefs of AI/AN people and their healthcare providers regarding e-cigarette use is important in the development and implementation of clinical preventive screening services for tobacco use and tobacco cessation treatment. Our primary research question was to describe sociocultural issues related to tobacco use and cessation to better interpret stakeholder understandings, preferences, and needs surrounding the use of pharmacogenetics (PGX) to guide cessation at a large tribally owned and operated healthcare facility. Within this healthcare system, AI/AN patients are known as customer-owners because, as tribal members, they own the healthcare system from which they receive services. Results on customer–owner, provider, and tribal leader views on tobacco use and the potential role of PGX to guide tobacco cessation treatment among AI/AN people are presented elsewhere.

The research results presented in this article describe emergent e-cigarette findings from the PGX interviews. In this article, we describe customer–owner, provider, and tribal leader views on e-cigarette use, screening for ENDS use, and role of ENDS in tobacco cessation treatment among an urban primary care setting serving an AI/AN population.

## Methods

This qualitative study used semi-structured interviews with customer–owners, providers, and tribal leaders to characterize perceptions of the utility of PGX to guide tobacco cessation treatment at Southcentral Foundation's (SCF) primary care clinics. The Anchorage Native Primary Care Center (ANPCC) is operated by the tribally owned SCF (22, 23). ANPCC provides comprehensive health services for more than 65,000 AI/AN people and operates through a pre-paid, patient-centred, medical home model (24, 25).

### Participant recruitment and data collection

The sample consisted of 3 stakeholder groups: (a) adult customer–owners with a current or past history of tobacco use; (b) providers (i.e. physicians, nurses, pharmacists, health educators, and dental hygienists) serving within the SCF primary care system; and (c) tribal healthcare system leaders (e.g. department managers within the SCF primary care system). All participants were over 18 years of age. Customer–owner inclusion criteria were AI/AN heritage, eligibility for services at the ANPCC, and either current tobacco use or a history of tobacco use. Healthcare providers and tribal leaders were affiliated with the ANPCC.

Customer–owners were recruited from the lobby of the ANPCC through posted recruitment flyers. Healthcare providers and institutional leaders were recruited via email from a list supplied by a project co-investigator. Recruits were screened to ensure eligibility requirements were met. Interviews were conducted in English at SCF from October 2013 through January 2014, digitally recorded, and transcribed verbatim for analysis. A brief demographic questionnaire was administered following each interview (Table I). Interviewers used a semi-structured interview guide (Table II) to complete interviews. The interview guide was designed to elicit rich descriptions of perceptions and practices. Interviews lasted an average of 30 minutes. A brief demographic questionnaire was administered immediately following each interview. Participants received a $25 gift card for participation. All digital data were stored on a password-protected computer server. Hard copies of questionnaires were stored in a locked cabinet in a secured room.

**Table I:** Participant characteristics

	Customer–owners	Leaders	Providers	All participants
Gender				
Female	14	9	10	33
Male	6	0	3	9
Age				
18–29 years old	7	1	0	8
30–39 years old	1	4	5	10
40–59 years old	9	4	8	21
60+ years old	3	0	0	3
Highest level education completed				
> High school	0	0	0	0
High school graduate	4	0	1	5
Some college	9	1	0	10
College graduate	3	5	10	18
Trade or vocational school	0	2	2	4
Past quit attempt (any type of tobacco)?				
Yes	16	5	5	26
No	4	4	8	16

**Table II:** Interview guide

Interview questions	Customer–owners	Leaders	Providers
*Views on tobacco use, consequences, and cessation*			
When did you start smoking or chewing? Has your tobacco use changed over time?	X		
Why do people use tobacco, in your view?	X	X	X
How does using tobacco affect a person's health and wellbeing, for better or worse?	X	X	X
What are the advantages and disadvantages of quitting tobacco?	X	X	X
*Experience with tobacco use and cessation at SCF*			
Could you share your personal tobacco story with me?	X		
What is your experience with quitting tobacco?	X		
Can you tell me how Southcentral Foundation (SCF) currently approaches tobacco use and cessation with customer owners?		X	X
In your experience, which tobacco cessation approaches are most useful/successful? Why do you think that is?			X
How do you approach tobacco use and tobacco cessation with customer–owners?			X
What questions do customer–owners tend to ask about tobacco cessation?			X
*Views on using pharmacogenetic testing for tobacco cessation at SCF*			
What comes to mind when you think about the phrase “genetic testing?”	X	X	
How do you feel about the use of genetic tests in your practice?			X
Let's say SCF wanted to participant in research that included genetic tests to understand how people respond differently to medications used for quitting tobacco – what would you think about that?	X	X	X
If SCF did this kind of research, would you participate? Why or why not? What questions would you have about it? What would you want to know about it?	X		
If SCF did this kind of research, how would you feel about your customer–owners participating?			X
What would you like your customers to know about genetic testing?		X	X
If a genetic test became available to help people choose the best medication for quitting tobacco, should SCF offer it? Why or why not?	X	X	X
If you had a customer–owner that was trying to quit tobacco and SCF offered a genetic test to guide your medication choice, would you order the test?			X
If you were trying to quit tobacco and SCF offered a genetic test to guide your medication choice, would you want to have the test? Why or why not? What other information would help you decide?	X		
How do you think customer–owners would respond to the use of genetic testing at SCF to guide tobacco cessation treatment?			X
*Concluding remarks*			
What else should we know about this topic?	X	X	X
How was this interview for you?	X	X	X

Many questions reworded to suite the role of the participant. Question follow-up probes were removed from interview guide for brevity.

### Human subjects and community review of research

Alaska Area Institutional Review Board approval and tribal approval were sought and granted prior to data collection. Participants provided limited demographic and health information; information was not linked during coding portion to better protect confidentiality. Demographic and transcript data were not linked during the coding portion of the analysis. Additionally, transcripts were cleaned to remove information that directly or indirectly identified a respondent. Finally, all resultant dissemination documents were reviewed by tribal review committees prior to dissemination.

### Data analysis

We conducted a thematic analysis of the interview data through an iterative and inductive process (26). A 3-person coding team began the analytical process by individually reading 3 sentinel interviews, one from each participant subgroup, and noting salient constructs in the margins. These initial constructs formed the basis of the team's consensus codebook. Sentinel transcripts were iteratively recoded until agreement was reached, yielding 20 initial codes. Using ATLAS.ti 7.1.8™, the team coded a subset of the remaining interview transcripts, meeting at the end of each week to re-establish code definition consensus, resolve discrepancies, and expand or collapse codes or definitions as needed. This process continued until all transcripts were coded and/or recoded as necessary. Primary themes were initiation and early tobacco use; cultural and social/situational uses of tobacco; perceived benefits and harms of tobacco use; drivers of successful cessation; pre-contemplative research views; existing research capacity and capacity building; and tobacco PGX research activity recommendations. These themes are described elsewhere. In the data analysis process, an emergent theme on e-cigarette use was found as participants described tobacco use, tobacco screening, and tobacco cessation. We present the emergent e-cigarette theme results here.

## Results

Demographic characteristics are reported in aggregate to avoid risk of participant identification. The sample consisted of 20 customer–owners, 12 primary care providers, and 9 tribal healthcare system leaders. As seen in Table I, the majority of participants were women (79%), over age 40 (58%), who had at least some college education (84%), had not participated in genetics research (85%; 8% unsure), and had attempted to quit tobacco at some time in their lives (65%).

As participants discussed tobacco use and tobacco cessation, participants from all sample groups independently commented on the emerging role of e-cigarettes as they considered questions on tobacco use harms and benefits, tobacco use change over time, and tobacco cessation approaches. Participants of all groups highlighted the novelty and popularity of e-cigarettes as an area of health concern, mentioned confusion about potential health benefits and health harm associated with e-cigarette use, and saw an opportunity for dialog between customer–owners and health providers as well as among healthcare providers. There were no differences in perception about e-cigarettes based on past quit attempt.

### E-cigarette advertisement

Evidence-based messaging on e-cigarettes was desired by participants in all 3 stakeholder groups to provide a counterpoint to the media messaging provided by e-cigarette vendors. One provider expanded on this idea, commenting,

What they're [customer–owners are] hearing from these people [vendors] are basically what they need to hear to sell the product. So they're not telling them about … anything associated with … the chemicals, and … any dangers with it. … helping them … be educated on that topic and get them thinking about … and see … what it really has in it.

### Variable e-cigarette use

Participants described how AI/AN people are using e-cigarettes. Providers stated e-cigarette use was most often seen among daily cigarette users rather than occasional or light smokers. Mixed use of both traditional cigarettes and e-cigarettes was seen among users of e-cigarettes. Use of e-cigarettes during specific activities of daily living (e.g. commuting) was noted. Many participants saw e-cigarette use as a lower risk way of consuming nicotine. For example, one smokeless tobacco-using customer–owner likened e-cigarette use to smokeless tobacco pre-packaged in single serving pouches. Participants pointed to the relatively low cost of e-cigarettes and e-liquids, the range of choices available, and the long-lasting nature of e-cigarettes as benefits of e-cigarettes compared to traditional commercial tobacco products.

### Screening for e-cigarette use

Provider and tribal leader participants mentioned concern that current tobacco screening practices within primary care settings were failing to detect or address e-cigarette use. The current practice for tobacco screening at SCF is for healthcare providers to ask, “Do you currently use tobacco?”. If a customer–owner responds affirmatively, he or she is asked, “Are you interested in quitting?”. One provider described a recent conversation with a customer–owner during tobacco screening that demonstrated the disconnect between the screening questions and e-cigarette use, commenting, “They're like, ‘Well, you know, I only smoke 3 cigarettes a day’, and I asked them, ‘Well, do you smoke any e-cigarettes?’ ‘Yeah, but that's not a cigarette’.”

### Role of e-cigarettes in clinical cessation

The role of e-cigarette use in tobacco cessation was questioned by administrative leaders, healthcare providers, and customer–owners. Tribal administrative leaders were unsure of SCF's formal stance on e-cigarette use in clinical tobacco cessation but were aware of standard nicotine replacement therapies such as nicotine gum, alternative drug treatments such as varenicline and bupropion, and counseling support options. One tribal leader stated, “I don't know if SCF dispenses electric cigarettes. I don't know if they'd be interested in it, but I know of several people who said, finally, this is a way that they can keep from lighting up.” Some customer–owners noted variability in cessation aid effectiveness and felt that e-cigarettes were a viable cessation aid option, noting that e-cigarette vendors often advertised the devices as cessation aids. One customer–owner described her decision to quit smoking for health reasons during her recent pregnancy: “… since I had the – the use of the e-cigarettes – you know, it was – it was actually easy for me to stop smoking for, you know, the time I was pregnant until the time I was nursing. I just started again just recently.” Finally, providers saw e-cigarettes as a substitute of one negative health behaviour for another. For example, one provider stated, “[I]f they quit regular cigarettes and [are] just smoking e-cigarettes, it's not really cutting a habit. You're still paying the cost …. And it's not … better for your health ….”

## Discussion

A lack of evidence-based information on the benefits and harms of e-cigarette use was apparent in all participant groups. Customer–owners displayed the most first-hand knowledge with e-cigarettes. The combination of customer–owners’ positive perception of e-cigarette use coupled with healthcare providers’ lack of knowledge of e-cigarette devices and use patterns may lead to customer–owner underreporting and providers not probing further for e-cigarette use when screening for tobacco use or treating nicotine addiction. If current preventive screening practices fail to identify or underreport individuals using ENDS, e-cigarette users may not be referred to clinical cessation services. This missed opportunity to support nicotine cessation may disproportionately affect AI/AN people.

Customer–owners who report higher consumption of traditional commercial tobacco products are more likely to use multiple nicotine delivery products (e.g. e-cigarettes in addition to tobacco cigarettes or smokeless tobacco) (27, 28). Thus, health benefits due to the reduction of tobacco use among AI/AN people through clinical tobacco treatment may be lessened among e-cigarette users. Presently, the SCF cessation treatment program is called the “Quit Tobacco Program” and its goal is for participants to abstain from tobacco use for life (11). As health systems consider adapting their screening, referral, and treatment programs to address nicotine use, the goal of treatment and the tracking of proximal and distal health outcomes will need reconsideration (15, 29–31). The health system will need to consider how e-cigarettes will be considered in the schema of tobacco products and harm reduction strategies to quit tobacco use.

This study had several limitations. Within this study, we did not explicitly ask about attitudes, perceptions, or behaviours regarding e-cigarettes and thus may have missed relevant information from participants. The AI/AN customer–owner participants were current or former tobacco users; among the former smokers, the time elapsed since quitting varied greatly in the sample, with some participants having quit well before e-cigarettes were developed and/or widely available. The sample was a convenience sample of AI/AN customer–owners, tribal leaders, and their healthcare providers and may not be representative of the views of these groups. The sample did not include individuals younger than 18 years; thus, the views of youth – a potentially important subgroup for e-cigarette use – are not represented in this sample. The sample included a preponderance of female participants, thus views of males may not be adequately reflected. Finally, saturation was reached on the primary research question of describing sociocultural issues related to tobacco use and cessation to better interpret stakeholder understandings, preferences, and needs surrounding the use of PGX to guide cessation; however, as e-cigarette use was an emergent theme additional study on the topic is warranted within this population.

Health systems need to rapidly update their screening, referral, database tracking, and cessation messaging to include health messaging and data collection systems that include e-cigarette use (32). Additionally, research on AI/AN cultural values around tobacco use and how those values are translated to e-cigarette use as health educators and other healthcare providers develop AI/AN-specific messaging on these novel products.
